# Neurolinguistics: Structure, Function, and Connectivity in the Bilingual Brain

**DOI:** 10.1155/2016/7069274

**Published:** 2016-01-05

**Authors:** Becky Wong, Bin Yin, Beth O'Brien

**Affiliations:** Education and Cognitive Development Lab, National Institute of Education, Nanyang Technological University, 1 Nanyang Walk, Singapore 637616

## Abstract

Advances in neuroimaging techniques and analytic methods have led to a proliferation of studies investigating the impact of bilingualism on the cognitive and brain systems in humans. Lately, these findings have attracted much interest and debate in the field, leading to a number of recent commentaries and reviews. Here, we contribute to the ongoing discussion by compiling and interpreting the plethora of findings that relate to the structural, functional, and connective changes in the brain that ensue from bilingualism. In doing so, we integrate theoretical models and empirical findings from linguistics, cognitive/developmental psychology, and neuroscience to examine the following issues: (1) whether the language neural network is different for first (dominant) versus second (nondominant) language processing; (2) the effects of bilinguals' executive functioning on the structure and function of the “universal” language neural network; (3) the differential effects of bilingualism on phonological, lexical-semantic, and syntactic aspects of language processing on the brain; and (4) the effects of age of acquisition and proficiency of the user's second language in the bilingual brain, and how these have implications for future research in neurolinguistics.

## 1. Introduction

It has been estimated that more than half of the world's population are bilinguals and/or multilinguals [1]. How does this widespread capacity for communicating in two or more languages impact the cognitive and brain systems in humans? For many years, the fields of psychology and neuroscience had limited tools and tended to investigate brain structure and cognitive function separately. However, recent advances in neuroimaging techniques and analytic methods have led to a proliferation of neuroscience findings regarding the impact of bilingualism on the human brain. Here we review these numerous and surprisingly diverse findings in light of current cognitive models, thereby enriching current understandings on the effects of bilingualism through mutual perspectives of linguistics, cognition, and neuroscience. Additionally, in the present review, we overcome the limited perspectives of early work in psychology and neuroscience by spanning the gap between brain structure and cognitive function. Specifically, we systematically examine the structural and functional differences in language networks for domain-general and domain-specific component processes in bilinguals/multilinguals (henceforth referred to as “bilinguals”: in this paper, we do not distinguish between findings pertaining to bilinguals versus multilinguals). We also focus on individual-difference factors including age of acquisition and language proficiency that may differentially impact bilingual brain networks. Throughout this review we argue that bilingual cognition is best understood by taking into consideration both structure and function, as well as factors relevant to language learning.

## 2. Historical Perspective

Early neuroscience perspectives on the relationships between brain structure and cognitive function drew two opposite conclusions from then-available cruder forms of investigation. Brain structure was considered to be organized into localized, isolated areas where pockets of activity serve very specialized function, as in the tradition of Gall and Spurzheim [2] and Fodor [3]. The alternative view was that brain structure is relatively homogeneous with distributed forms of representation, as in the tradition of Lashley [4] and Hebb [5]. According to this view, brain structure/architecture was related to function in a more holistic way that supports plasticity, whereby functions associated with damaged areas can be picked up by other undamaged areas. The former perspective was supported by myriad functional neuroimaging studies (e.g., [6–8]), while the latter perspective was taken up by connectionist investigations [9–11].

Driven by new technologies and advances in computing power, contemporary approaches to neuroscience have now been able to draw evidence from more sources than in the past, leading to new perspectives placed between these two extremes. Thus, the new evolution of neuroscience investigations is not structurally bound, like the past lesion studies, nor functionally discrete, like the early neuroimaging studies, but can accommodate both perspectives of local specialization with global coordination across areas as self-organized networks that emerge from the individual's experiential history. Current neuroscience-based models can therefore point to a developed neural substrate of networks forged by nature and nurture, which instantiate soft-assembled coordinative structures [12] organized to meet the constraints of the current behavioral task.

As a case in point, language is multifaceted, with oral and written forms as well as receptive and expressive modes, but shows evidence of certain universal properties of brain structures and their interconnections that underlie the behavioral aspects of communicating through speech and/or print in multiple languages. While structure gives clues to the architecture of language networks, function relates to the manner in which networks may be assembled within different contexts or as a result of personal experiences. At present, unresolved questions for bilingualism include the degree to which there is anatomical overlap in the neural networks used for L1 and L2 processing in various language domains.

One possibility is that there is a common neurobiological foundation for different languages (e.g., [13]), addressed below as a “universal language neural network.” Although brain networks may be highly constrained across languages and routed to the same cortical circuits [14, 15], this explanation may not be tenable in the case of bilingualism, given that anatomical overlap between language networks could be sensitive to variables such as language proficiency, age of acquisition, and different scripts. A second possibility is that the spatial organization of the neural networks for acts of reading, listening, and speaking is common across one's different languages, only to the extent that a high proficiency is reached for the languages in question. This would indicate that the universal neural network is only accessed at the end of the L2 learning process. Alternatively, a third possibility is that the linguistic brain structures established from L1 acquisition are coopted for L2 learning only during a critical window of chronological development; that is, only early age of L2 acquisition or simultaneous bilingualism would allow for the assimilation of a universal neural network across languages.

In the next section, we briefly introduce advanced neuroimaging techniques and paradigms that have allowed researchers to investigate the impact of bilingualism on brain structure, structural connectivity/physical coupling, brain function, and functional connectivity/statistical coupling.

## 3. Neuroscience Methodologies

### 3.1. Techniques Used to Investigate Brain Structure

Magnetic Resonance Imaging (MRI) is a neuroimaging technique that produces high quality images of the internal structure of the living brain by using magnetic fields and radio waves to detect proton signals from water molecules [16]. It provides structural information such as neural volume (total brain volume, gray matter, and white matter volume), cortical thickness, and surface area [17]. Voxel-based morphometry (VBM) is an analysis technique that uses T1-weighted MRI scans and performs statistical tests to identify differences in brain anatomy [18].

### 3.2. Techniques Used to Investigate Structural Connectivity

Diffusion Tensor Imaging (DTI) is another technique that makes use of the MRI machine to image the neural tracts and fibre pathways that connect brain regions, so as to gauge thickness and density of axonal connections through measures such as fractional anisotropy [19]. Diffusion Spectrum Imaging (DSI) goes one step further in that it was specifically developed to image complex distributions of intravoxel fiber orientation, so as to overcome the inability of DTI to image multiple fibre orientations [20].

### 3.3. Techniques Used to Investigate Brain Function

Functional Magnetic Resonance Imaging (fMRI) detects the magnetic signal resulting from blood oxygenation and flow that occur in response to neural activity [21, 22]. Functional near infrared spectroscopy (fNIRS) is an optical neuroimaging method that goes beyond fMRI in that the latter simultaneously measures the changes in oxygenated, deoxygenated, and total haemoglobin, is portable, and can be used for both children and infants [23, 24].

Electroencephalography (EEG) measures cortical electrical activity by recording from electrodes placed on the scalp [25]. Researchers typically examine the electrical waveforms for their frequency (e.g., alpha, beta, delta, and theta), intensity and timing, typically seen in event-related potential (ERP) components, and signal coherence/synchrony [26].

Magnetoencephalography (MEG) allows researchers to study neural function in real-time based on the magnetic fields produced by neural electrical activity and, like EEG, is a technique with good temporal resolution [27].

### 3.4. Techniques Used to Investigate Functional Connectivity

Psychophysiological interactions analysis (PPI) is a method for investigating functional connectivity between different brain areas using fMRI data [28]. Effective connectivity [EC] analysis studies the causal influence that one neural system has on another using fMRI data, so as to allow for a richer understanding of interregional brain connectivity [29, 30].

The review that follows is split into three sections. First, we provide an overview of a “universal” language network across languages and examine how this network functions in bilinguals. We posit that, upon learning a second language (L2), some of the same structures are engaged, such that first language (L1) and L2 processing show similar patterns of activation across these brain networks (e.g., [31–34]). Additionally, areas besides those closely associated with language function are drawn into the processing of multiple languages and some are correlated with the switching between languages [35, 36]. In the second section, we focus on the specific brain areas and subnetworks that are related to three key aspects of linguistic processing, namely, phonological, lexical, and syntactic components of linguistic processing. Finally, we consider variations to the universal language network in general and to the linguistic component subnetworks in particular, in response to variables of age of acquisition and language proficiency.

## 4. A Universal Language Neural Network

There is compelling evidence for a “universal” language network of the human brain. Initial insights into this network, from lesion studies, put forth the classical perisylvian language network, consisting of Broca's area (BA44) in the inferior frontal lobe, Wernicke's area (BA22) in the superior posterior temporal lobe, and the arcuate fasciculus connecting the two [37], all left lateralized in most individuals. Additionally, acts of speech draw on the caudate nucleus, superior frontal gyrus, and superior longitudinal fascicle (SLF) [38]; and acts of reading draw on visual association areas, fusiform gyrus, and the angular gyrus [39].

Among languages, including distant ones like Mandarin and English, identical areas of activation are found for speech production tasks. Word generation and rhyming tasks elicit equal activation in L middle frontal cortex and L inferior prefrontal gyrus for both Chinese and English rhyming [40], prefrontal, temporal, and parietal areas, and the supplementary motor area for English and Mandarin word generation [41]. The results of a meta-analysis of 24 studies on word production found no significant differences in hemodynamic activation between L1 and L2 processing on a variety of experimental tasks [42].

With regard to listening, equivalent areas of activation, including the L temporal pole, the superior temporal sulcus, middle temporal gyrus, and hippocampal structures, are found for bilingual individuals in both L1 and L2 [43, 44]. These findings are in line with connectionist theories which see the language network as a single system, with L2 learning being a matter of simply increasing the strength of certain connections within the same network [45, 46].

Across various languages, a common reading network is engaged. This includes dorsal, anterior, and posterior ventral systems [13, 47]. The dorsal system includes the angular gyrus and posterior superior temporal gyrus and is associated with mapping orthography onto phonological and semantic information. The anterior system includes the posterior inferior frontal gyrus and is related to decoding new words. The ventral system, including the left inferior occipitotemporal area, functions as a presemantic word form area.

Each of these linguistic acts (speech, listening, and reading) engages some common areas and requires knowledge represented at different linguistic components, including information about sound structure (phonology), word based meaning (lexical, vocabulary), and word integration (syntax). Given results from behavioral studies, the assemblages of subnetworks related to these different components of linguistic knowledge may show some departure from the common network for bilinguals. Tasks related to these component processes of language have been correlated with activity in the following brain structures.

*Phonology*. Phonology draws on the auditory input system in Heschl's gyrus [38], auditory association areas in the perisylvian region, including superior temporal gyrus, inferior parietal cortex, inferior frontal gyrus, and the arcuate fasciculus-Broca's area-Wernicke's area pathway [37].

*Semantic Vocabulary Knowledge*. Semantic vocabulary knowledge draws on amodal association areas such as the middle temporal gyrus, posterior STS, temporoparietal cortex, supramarginal gyrus, anterior inferior frontal cortex, a long-range dorsal fibre tract that connects the temporal lobe with Broca's area, and also the angular gyrus for sentence-level semantics [38, 48].

*Syntax*. Syntax draws on the L pars opercularis, pars triangularis in Broca's area, and the posterior superior temporal gyrus, connected by the arcuate fasciculus [38, 49].

While the general language network may be similar across languages and even between languages used within a bilingual individual [33, 50–55], there appear to be more variations in the way these subnetworks for the component processes are engaged and assembled. This may partially result from certain features of bilingualism that differentially impact the way that two or more languages are managed. In particular, the age at which one learns a second language affects whether these subnetworks overlap or utilize separate brain areas, implying that language learning is neurophysiologically instantiated in a different manner across development (e.g., [56]).

Further, language proficiency is also differentially related to both structure and activity across brain areas, indicating a similar modification of the way language is instantiated in the brain over the course of learning the language (e.g., [52]). These two factors, age and order of language acquisition plus achieved proficiency, are partially associated and may interact with the overlap versus divergence of neural pathways used for language tasks, such as reading. For instance, simultaneous acquisition of reading in two orthographies lends itself to divergent pathways for reading in each language, whereas sequential reading acquisition gives rise to largely overlapping reading circuits in both languages [57].

In some cases, the type of language (tonal versus nontonal; or logographic versus alphabetic orthographies) involved in bilingualism also results in variation on structural and functional differences in the brain [47, 58, 59]. One area of difference in brain circuitry/function for bilingual compared with monolingual individuals pertains to executive function processes, as described next.

## 5. What Are the Effects of Bilinguals' Executive Functioning on the Structure and Function of the “Universal” Language Neural Network? Do These Effects Differ from Those of Monolinguals, and If so, How?

The three core aspects of executive functioning that have been identified by Miyake et al. [60] are inhibitory control, shifting, and updating. Inhibitory control refers to the ability to deliberately override a dominant or automatic response [61, 62]. Shifting refers to the ability to move flexibly between multiple tasks or operations [60, 63]. Updating refers to the ability to monitor information that is held in working memory and revising it as appropriate with newer or more relevant information [64, 65].

Many behavioral studies and reviews have found that bilinguals show advantages in tasks of executive functioning (EF) (e.g., [66–68]). EF tasks require the participants to engage in high-level cognitive functions to coordinate their thoughts and actions for goal-directed behaviors [69–71]. It must be noted, however, that this argument of a bilingual advantage in EF has recently been under much scrutiny and debate (e.g., [72–74]). For example, Paap et al. [75] argue that there have been possible methodological issues with and differences among behavioral studies, involving inappropriate baselines and/or questionable use of statistical tests.

In this section, we contribute to the ongoing discussion by examining theoretical models for bilingual language processing and empirical findings from neuroscience studies on the structural, functional, and connective changes in the human brain that ensue from bilingualism and whether this differs from monolinguals, in an effort to clarify the “hazy” differences between bilingual and monolingual brains [72].

Cognitive models of bilingual language processing implicate a specific role for nonlinguistic executive functioning [76]. For example, Green's [77] inhibition control model posits that bilinguals experience a constant competition between the lexical representations of both languages and therefore must use inhibitory control—a domain-general resource—to inhibit the activation from the nontarget language. Similarly, the bilingual interactive activation+ model [78] proposes that there is a decision and response selection mechanism that imposes top-down control in selecting between activated lexical representations. Some consensus has emerged in the literature that bilinguals recruit some measure of domain-general executive control to switch between languages [79, 80]. These models strongly suggest the importance of executive functioning in language processing for bilinguals.

It must be noted, however, that there may be a difference between executive functioning for bilingual language control (e.g., switching between languages and/or inhibiting nontarget lexical representations) versus nonlinguistic executive functioning (e.g., switching between tasks). Preliminary research indicates that bilinguals' advantage for executive functioning might be limited to the former ([79, 81], cf. [82]).

Moving on to the effects of executive functioning and language control on the bilingual brain, neuroscience studies have found structural, functional, and connectivity differences in brain areas associated with domain-general cognition for bilinguals as compared to monolinguals, particularly in the basal ganglia and the frontoparietal brain network.

### 5.1. Structure

Studies have found differences in brain structure between bilinguals and monolinguals particularly in frontoparietal brain areas traditionally associated with cognitive control and executive functioning. For example, using voxel-based morphometry (VBM), Mechelli et al. [83] found that grey matter density in the inferior parietal cortex was higher in bilinguals than monolinguals and that this effect was sensitive to age of acquisition and proficiency. Specifically, the structural difference was more pronounced in early bilinguals than late bilinguals, as well as bilinguals with a higher L2 proficiency. Using high resolution anatomical MRI, Della Rosa et al. [84] also found that multilingual children had greater grey matter density than monolingual children in the inferior parietal lobe. The authors argued that increased grey matter in the IPL was likely the source of their enhanced attentional and cognitive control.

A number of researchers have also found structural differences in the basal ganglia, particularly the caudate nucleus. For example, using VBM, Zou et al. [85] found that grey matter volume in the left caudate nucleus was higher in bilinguals than monolinguals. The researchers argued that this area was implicated in cognitive control, because functional activation in the caudate nucleus was higher when bilinguals switched between languages, compared to when they did not switch. Hosoda et al. [86] reported that a training intervention for L2 vocabulary in bilinguals resulted in a significant increase in grey matter volume in the caudate nucleus, among other brain areas. A review by Li et al. [87] similarly reported that bilinguals consistently show greater GM volume and density in the caudate nucleus as compared to monolinguals.

Given that the basal ganglia, particularly the caudate nucleus, is known to be part of an anatomical network subserving functions within the dorsolateral prefrontal cortex [88] for goal-directed behavior [89], and since this brain area is implicated in switching between languages in the bilingual brain [90], it is plausible that this brain area, together with the frontoparietal network, might be the locus of the bilingual advantage in executive functioning and language control.

### 5.2. Structural Connectivity

Using DTI, Grady et al. [91] found stronger connectivity in the frontoparietal control network in bilinguals compared to monolinguals when they were at rest. This network includes the dorsolateral and inferior frontal regions and the inferior parietal lobe and is well-known to be implicated in executive functioning, attention, and cognitive control [92].

### 5.3. Function

Using fNIRS, Kovelman et al. [93] found that bilinguals activated the dorsolateral prefrontal cortex (DLPFC) and the inferior frontal cortex in a semantic judgment task more strongly than did monolinguals, even though both groups had equally good performance in the task. Since previous research has linked the DLPFC with working memory [94, 95], the researchers took this to mean that the ability to process more than one language might have led to functional changes in brain regions that support working memory associated with language processing.

**Figure 1:**
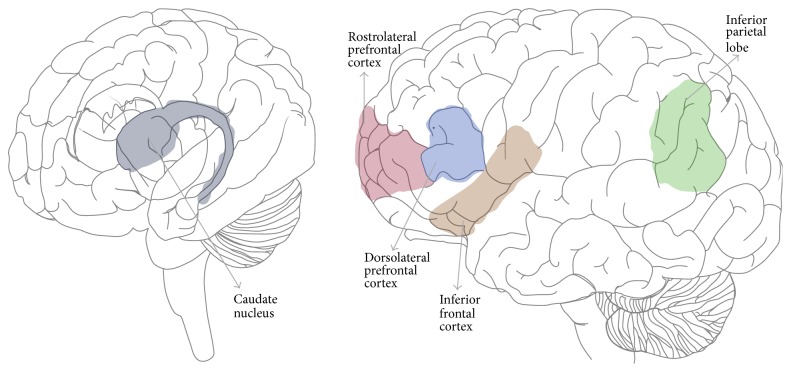
Key brain areas implicated in executive functioning in bilinguals.

This parallels the findings of a fNIRS study by Jasińska and Petitto [96], where the researchers found that bilinguals activated the classic left hemisphere language areas (L inferior frontal gyrus, superior temporal gyrus) and the domain-general cognitive areas (dorsolateral prefrontal cortex, rostrolateral prefrontal cortex) more strongly than did monolinguals in a reading task. Previous research has found that RLPFC is linked to planning, reasoning, and integrating information [97, 98], and the DLPFC to working memory. As such, the researchers proposed that the bilinguals' experience with monitoring and selecting between two language systems might have been linked to the greater prefrontal cortical activation. The key brain regions implicated in executive functioning in bilinguals as compared to monolinguals are shaded in Figure 1.

In light of the recent debate on the bilingual advantage in EF, then, while we acknowledge that the frequency and effect size of the bilingual advantage in EF may have been inflated by questionable practises among behavioral studies, we argue that existing neuroscience findings paint a consistent picture of the bilingual advantage in EF. Certainly, higher numbers of participants would be ideal for future neuroscience studies to increase statistical power (cf. [72, 73]). However, at our current state of knowledge, current research points to stronger EF in bilinguals, along with increased gray and white matter volume and regional activation in areas associated with cognitive control (specifically, frontoparietal network and basal ganglia, as shown in Figure 1), supporting the notion of a bilingual advantage in executive functions.

Yet another set of findings indicates a bilingual* disadvantage* in specific language abilities/competencies. Friesen and Bialystok [99] frame this disparity in terms of control mechanisms versus representations, or crystallized knowledge. They make the case that while the control mechanisms, such as cognitive control and executive functioning outlined above, seem to be better for bilinguals than monolinguals, the opposite is true for lexical representation. Behaviorally, monolinguals display faster picture naming times than bilinguals in either language [100, 101] and produce more words in verbal fluency tasks [102], particularly in the initial portion of such timed tasks [103]. More specifically, a bilingual disadvantage holds for some forms of crystallized knowledge, such as vocabulary knowledge (e.g., [104]), but not for others, such as metalinguistic skills like phonological and morphological awareness (e.g., [93]).

## 6. How Is the Differential Effect of Bilingualism on Phonological, Lexical-Semantic, and Syntactic Aspects of Language Reflected in the Structure and Function of the “Universal” Language Neural Network?

Cognitive models help to differentiate these knowledge forms or representations of language further. For instance, Ullman [105, 106] differentiates language processes that involve more declarative memory from more rule-like aspects of language. Vocabulary knowledge and word phonology both involve arbitrary mappings between word labels and their meanings and would draw on declarative memory. Syntax and grammatical knowledge would constitute procedural forms of memory that involve rule-based learning instead of arbitrary mappings.

Kroll and Stewart's [107, 108] model of second language learning makes the further distinction between declarative memory as word labels and concepts or semantic knowledge. In their model, the former consists of two separate lexical stores, with one store of word labels per language, and the latter is a single store of word meanings that become linked to the word labels from both language lexicons. Word labels are essentially the arbitrary sounds or printed forms associated with the lexical entry and constitute the whole word phonology. For a bilingual, these arbitrary links between lexical labels to semantic features are said to be weaker [109].

How do these cognitive models play out in the brain? Taylor et al.'s [110] meta-analysis of reading studies using neuroimaging found support for Ullman's [105, 106, 111] model with regard to declarative, temporal lobe versus procedural, cortical-subcortical systems function-to-structure links. They reported clusters of activity related to various language tasks, all in the left hemisphere. Cluster-to-function relations included the occipitotemporal cortex related to orthographic analysis; the anterior fusiform and middle temporal gyrus related to lexical/semantic processing and declarative memory; the inferior parietal cortex related to spelling-sound conversion and procedural memory; and the inferior frontal gyrus as related to phonological output resolution and procedural memory.

Thus, while many of the same areas and networks are utilized for both L1 and L2 in bilinguals, even within these shared regions there may be differences in the level of activity (e.g., fMRI) and the degree of connectivity (e.g., DTI) in bilinguals compared with monolinguals. We might expect that relative weakness in semantic/lexical representations (e.g., [104]) and syntactic knowledge (e.g., [112, 113]) for bilinguals is reflected in structural differences as less gray matter volume and white matter connectivity, and functional differences as less activation in areas and subnetworks that are related to lexical and syntactic processing. On the other hand, subnetworks related to metalinguistic processing, such as phonology, may show structural and functional differences in the opposite direction in bilingual individuals, that is, increased gray and white matter and increased activation (following [93, 114]).

In the next sections, we review structural and functional integrity of the respective neural areas and systems related to phonological, lexical, and syntactic processing for bilinguals compared with monolinguals and within-individual differences in processing one's L1 versus L2. In contrast to the universal language network noted above for linguistic acts generally, we show that neural structure and function for these linguistic components are affected by one's linguistic experiences.

### 6.1. Phonological Processing

Phonological awareness is a metalinguistic skill, which involves the awareness of and ability to discriminate or identify phonological structure of one's language. This includes knowledge of rhyme, syllables, and phonemes (i.e., the smallest units of speech, such as* ba, da*). In alphabetic languages, phonological awareness is a foundational skill for literacy acquisition and also contributes to learning to read nonalphabetic languages [115]. Bilingual exposure facilitates superior phonological awareness [116]. The focus of this section is on brain areas involved in phonological processing for bilinguals and L2 learners.

#### 6.1.1. Structure

Overall, there is evidence for significant differences in brain structure between bilinguals and monolinguals across many of the traditional language areas, including those areas related specifically to phonology. Gray matter volume (VBM) is reported to be significantly greater in bilinguals for Heschl's gyrus [117], the superior temporal gyrus [118–120], left inferior parietal cortex [83, 121, 122], and inferior frontal areas [123].

Functionally, monolingual studies indicate that the superior temporal gyrus is linked to acoustic and phonological processing, while the inferior parietal cortex is linked to semantic/lexical learning [87]. Additionally, increased grey matter volume is found in the caudate nucleus [85], which is associated more with phonemic than semantic fluency [33].

#### 6.1.2. Structural Connectivity

Structural connectivity differs between bilinguals and monolinguals as well. For instance, DTI studies using fractional anisotropy (FA) report that superior bilingual language ability is linked to greater white matter in the arcuate fasciculus [124] and temporoparietal connections [125]. Luk et al. [126] also report higher FA values for early bilinguals compared to monolinguals, for tracts in the right inferior frontooccipital fasciculus, uncinate fasciculus, and the superior longitudinal fasciculus. The arcuate fasciculus connects the temporal cortex with the pars opercularis region of Broca's area (BA 44), and the superior longitudinal fascicle (SLF) also connects superior temporal gyrus to the premotor cortex [127]. The uncinate fasciculus links the anterior temporal lobe with the inferior frontal gyrus, whereas the frontooccipital fasciculus links the frontal lobe to the occipital lobe, as the name implies.

#### 6.1.3. Function

Functional imaging and electrophysiology studies also show variations in activity patterns across similar areas for bilinguals. ERP studies demonstrate that in adults and infants perception of speech phonemes is categorical. For example, there are sharp boundaries between perceiving an acoustic signal as a/p/versus a/b/. This is shown with a mismatch negativity paradigm, and has been localized to the left planum temporale, posterior to the auditory cortex [128].

Similar studies with bilingual participants show that second language speakers of a language also demonstrate this categorical phoneme perception. Using near-infrared spectroscopy (fNIRS), Minagawa-Kawai et al. [129] reported categorical phoneme perception of Japanese vowels in groups of both Japanese L1 and L2 speakers. However, the L2 group showed slower response times, and only the L1 group showed a relation of performance to activity in the left auditory area. Further, Tan et al. [40] found similar activation across L1 and L2 (Chinese and English) on a rhyme task related to L middle frontal cortex and L inferior prefrontal cortex. The authors concluded that the bilinguals used similar phonology networks and transferred their syllable level processing from L1 to L2.

Even in infancy, activation of areas related to a phonetic discrimination task has been found to be similar between monolinguals and bilinguals [130]. Both groups activate parts of the language network also found in adult studies, including the L superior temporal gyrus (related to phonetic processing in adults) and the L inferior frontal cortex (related to semantic retrieval, syntax, and phonological patterning in adults).

In their study with bilingual adults, Grogan et al. [33] established a link between function and structure with regard to phonological processing for speech production. The authors found that participants with better phoneme fluency than semantic fluency had increased grey matter density in the bilateral presupplementary motor area and the head of the caudate. Importantly, this positive relation of function and structure was strongest for the bilinguals' L2 compared with their L1.

Given the purported role of the caudate nucleus in procedural memory [111] and control processes [90], this finding demonstrates how processing of a second language impacts brain circuitry beyond language areas. In this case, the caudate may be drawn upon to manage activation and suppression between the bilingual's lexicons and to assemble the phonological sequence for articulating a response in the targeted language.

#### 6.1.4. Functional Connectivity

Using psychophysiological interaction analysis in English and Chinese pseudoword rhyming tasks, Cao et al. [47] found differences in functional connectivity during L1 and L2 processing. In the L1 tasks, greater connectivity occurred in the R precentral gyrus and three visuo-orthographic regions (L fusiform gyrus, L middle occipital gyrus, and R middle occipital gyrus), suggesting active sensorimotor processing during Chinese word rhyming. In the L2 tasks, greater connectivity occurred between the L postcentral gyrus and the R middle occipital gyrus, suggesting the importance of somatosensory feedback for this task with foreign phonemes.

Using graph theory in their study of bilingual and monolingual adults, García-Pentón et al. [131] reported two main networks that show stronger connectivity in bilinguals than monolinguals. The first comprises L frontal, parietal, and temporal regions (insula, superior temporal gyrus, pars triangularis and pars opercularis of the inferior frontal gyrus, and medial superior frontal gyrus). This network is potentially involved in phonological, syntactic, and semantic interference between languages. The second network involves the L occipital and parietal-temporal regions (R superior frontal gyrus, L superior occipital gyrus, R superior frontal gyrus, L superior parietal gyrus, L superior temporal pole, and L angular gyrus). This second network is postulated to facilitate visual word recognition, reading, and semantic processing.

Both of these networks were more graph-efficient in bilinguals as compared to monolinguals; that is, they had higher capability of transferring information, as higher efficiency indicates that pairs of nodes “have short communication distances and can be reached in a few steps” [132, page 14]. Further, age of language acquisition also played a role, whereby early acquisition resulted in the development of specialised structural brain networks in terms of higher connection density between regions and more graph-efficient flow of information.

In sum, across the structural, functional, and connectivity investigations, phonological processing areas show some differences between bilinguals compared to monolinguals. As predicted, bilinguals showed increased brain volume in traditional phonology-related areas (temporal, temporoparietal, and frontal areas) and greater connective white matter volume between these areas (AF, SLF, and uncinate fasciculus). At a basic level, these structural differences may correspond to the enhanced phonological awareness that comes with bilingual exposure, supporting the hypothesis that areas related to spelling-sound conversion (IPC) and phonological output (IFG) would show increases in structure and function with bilingualism (following [93, 114]).

Functionally, bilinguals' ERPs were qualitatively similar to monolinguals' for phoneme perception tasks, even though overt behavioral responses were slower and unrelated to temporal brain area activity. This might indicate weaker declarative-knowledge types of representations that may not be consolidated within the temporal brain areas. On the other hand, frontal area activation (L IFC) continued to be elicited with phoneme discrimination by bilingual infants for both L1 and L2 as they grew older (12 months), whereas this frontal engagement for a second language dropped out for monolingual infants. Also, performance on L2 speech production tasks requiring phonemic processing was positively related to increased structural volume in frontal (SMA) and basal ganglia (CN) areas.

**Figure 2:**
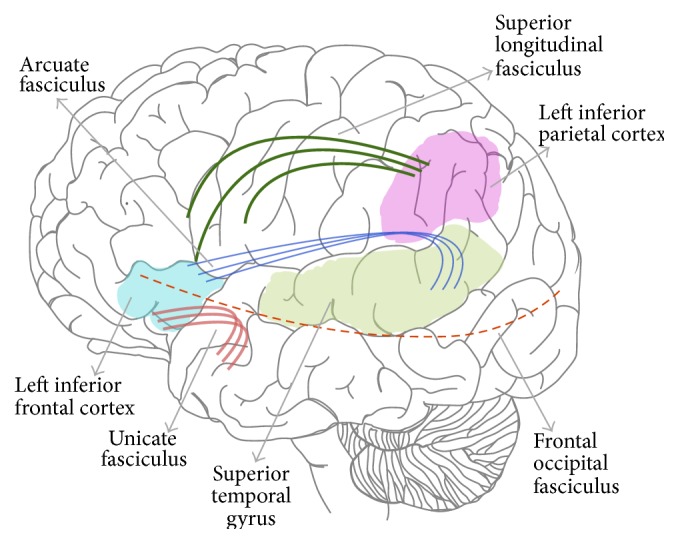
Key brain areas and connections showing variation in structure and functional activity for bilinguals performing phonological processing tasks.

With regard to connective networks, bilinguals also showed different assemblages for each language when making rhyme judgements and overall greater estimated processing efficiency within local subnetworks (frontoparietotemporal and occipitoparietal). At the same time, they evidenced less global whole brain network efficiency. These findings suggest that bilingualism may result in the formation of early specialized subnetworks that deal with phonological, as well as semantic and syntactic, information between languages [131]. The key brain areas and connections showing variation in structure and functional activity for bilinguals performing phonological processing tasks are illustrated in Figure 2.

### 6.2. Lexical-Semantic Processing

Lexical knowledge encompasses both the breadth and depth of the meaning of words, where breadth indicates the number of known words or vocabulary size, while depth indicates the degree of representation of a known word, including its semantic connections to other words (synonyms, antonyms), and morphological and syntactic variations [133]. Vocabulary bears strong relations to reading comprehension, directly and indirectly through conceptual knowledge [134]. Both vocabulary breadth and depth are reduced in bilinguals' languages [104, 135]. The focus of this section is on neural correlates of lexical-semantic processing by bilinguals and L2 learners.

#### 6.2.1. Structure

For monolinguals as well as bilinguals, MRI studies reveal that vocabulary size correlates positively with grey matter volume in the L and bilateral supramarginal gyrus in the left hemisphere [136–138]. As a group, bilinguals show greater volume in these areas compared with monolinguals [83, 139].

Grey matter volume in the L pars opercularis of the inferior frontal gyrus is also positively related to speed and accuracy of making lexical decisions, verbal fluency [139], and expressive vocabulary [86].

#### 6.2.2. Structural Connectivity

Studies investigating structural connectivity in lexical-semantic processing similarly implicate supramarginal areas and the IFG. For example, using fractional anisotropy (FA) analysis, Hosoda et al. [86] found that white matter between the IFG-pars opercularis and the supramarginal gyrus and the superior temporal gyrus is related to increased L2 competence. This relation was stronger in the R hemisphere structures and increased after L2 vocabulary training, along with increased connectivity in the R pars opercularis-caudate nucleus pathway. This plasticity was transient, however, and reversed at one-year follow-up [86].

#### 6.2.3. Function

While some findings with functional imaging show overlap in areas activated in bilinguals and monolinguals for lexical-semantic tasks, others show that some disparities exist as well. As with the structural studies, the two main sets of findings pertain to the frontal cortex and temporal cortex.

For frontal areas, Kovelman et al. [140] found that early bilinguals showed different brain activation patterns compared with monolinguals in the prefrontal cortex (DLPFC and IFC), even though they recruited similar language areas (Broca's 44/45). These differences occurred when bilinguals had to use both or either of their languages. When bilinguals had to use one language only, they showed greater signal intensity (as measured by changes in oxygenated hemoglobin) in DLPFC and IFC areas. This finding was taken to suggest neural activity to support working memory and attention associated with bilingual processing.

Similarly, in a visual lexical decision task with morphologically related primes, Bick et al. [141] found that highly proficient Hebrew-English bilinguals activated the L inferior and middle frontal gyri and occipital-temporal regions regardless of language type. However, the degree of activation was modulated by semantic properties for English only, showing cross-language sensitivity to differences in linguistic structure.

Vingerhoets et al. [142] reported that late multilinguals show similar regions of activation regardless of language used (Dutch, French, and English) during covert lexical-semantic processing. However, certain task-specific requirements activated additional areas during L2 processing. Specifically, picture naming involved additional L2 recruitment of L frontal areas, and inferior frontal, lateral, and medial areas (including Broca's), while word generation involved additional recruitment of inferior frontal and L middle temporal gyri for L2 processing.

Yet other studies report differences in functional activation in the temporal cortex for L2 lexical-semantic processing. Jeong et al. [143] manipulated whether L2 Korean words by Japanese learners were learned through situation-based dialogue or from print. They found that the R supramarginal gyrus was active for L2 words learned in the former manner, while the latter manner of learning drew greater activation in the L middle frontal area (WM) during the retrieval test. Further, when words that were learned in one condition were tested in the other condition (e.g., situation-learned, print tested), the L inferior frontal gyrus was activated, supporting the role of IFG in flexible retrieval of L2 vocabulary.

Raboyeau et al. [144] examined fMRI activation patterns during phases of learning new L2 Spanish vocabulary by French speakers (early, first 5 days, and later, 2 weeks). Left inferior frontal and Broca's region activity was associated with early learning, along with anterior cingulate cortex and DLPFC activation, suggesting the role of these areas in effortful lexical retrieval, phonological output, and monitoring, respectively. During the extended learning phase, L premotor cortex and R supramarginal gyrus as well as cerebellum areas were activated.

Finally, Crinion et al. [145] found with a semantic priming task in German-English and Japanese-English bilinguals that L ventral anterior temporal lobe activity was reduced with semantic primes (compared with unrelated primes) regardless of the language and regardless of whether the prime and target were in the same language. In contrast to this language general effect, a whole brain fMRI analysis found language-specific effects in the L head of the caudate nucleus. In this case, only semantically related word pairs that were presented in the same language showed reduced activity; other conditions with different language pairs showed increased activity in the CN. This suggests a role of the CN in lexical-semantic control, which the authors interpreted as a possible mechanism for regulating output given variations in language input.

#### 6.2.4. Functional Connectivity

Ghazi Saidi et al. [146] examined functional connectivity after L2 vocabulary training for Persian-French bilinguals in a picture-naming task. They reported increased functional connectivity within both networks with increasing L2 proficiency. The two networks included language areas (L temporal, perisylvian, and frontal areas) on the one hand and domain general cognitive control areas (bilateral cingulate, postcentral gyri, R superior parietal and inferior frontal gyri, and L superior frontal gyri) as regions of interest on the other.

In sum, lexical-semantic processing in bilingual and L2 learners is associated with similar areas as in monolinguals and L1, including anterior inferior frontal cortex and supramarginal gyrus. Increased volume and structural connectivity between these areas is reported for bilingual and L2 learners. Additionally, structural connectivity between inferior frontal with superior temporal gyrus, as well as the caudate nucleus, is also related to training induced changes in L2 vocabulary. Activation in these areas of the temporal and frontal “universal reading network” also showed increases in bilinguals or L2 learners when they process lexical-semantic information. While most areas were insensitive to different scripts, there was some indication that task type or learning modes or phases impacted different parts of the network, with frontal areas (e.g., L IFG) relating to early learning phases and flexible retrieval (across modes) of new vocabulary in an L2.

In light of behavioral findings that point to a bilingual disadvantage in lexical knowledge, we had predicted generally lower structural volume and connectivity, as well as lower function-related activity and connectivity for bilingual and L2 lexical-semantic processing. Yet this is not the pattern of the reported results. Instead, we observe apparently divergent findings between structure and function for bilinguals on one hand and behavioral differences between bilinguals and monolinguals on the other. The set of results here, taken in line with the behavioral findings of reduced bilingual lexical knowledge and efficiency, may need to be considered in light of effortful versus efficient processing (e.g., see [110]). In other words, reduced knowledge and efficiency observed behaviorally may be reflected neurally in terms of more volume and activation, characteristics of more effortful processing. This may be the case for the studies examining function, where bilingual compared with monolingual groups showed greater activation during semantic word processing tasks, especially in the prefrontal and inferior frontal cortex—areas outside the language circuits. These areas correspond more closely with general cognitive functions like working memory and may therefore reflect greater effort for bilinguals even when only processing one of their languages (e.g., [93, 114]).

With regard to the structural findings, both gray and white matter volume were greater for bilingual groups and even more so for multilinguals, possibly as a correlate of overall vocabulary size across known languages. These metrics also waxed and waned with second language proficiency after L2 vocabulary training. This suggests a more fluid relation of structure and function compared with our original hypothesis, and the findings above further suggest that the neural substrate assembled for lexical-semantic processing is responsive to both context-specific factors (e.g., [143]) and language-specific contexts (e.g., [145]).

### 6.3. Morphosyntactic Processing

In this section, we discuss the neural correlates of syntactic representation and processing in bilinguals/L2 learners. Syntax is a module of grammar which can be defined as a system of combinatorial rules that enables the generation of an infinite number of sentences from a finite lexicon. Syntactic knowledge can be characterized by its generative and systematic nature. The rule-based nature of syntax is in contrast with vocabulary, where the form-meaning association in words (i.e., lexical knowledge) is largely arbitrary. As mentioned above, these two types of knowledge are thought to be acquired via different memory systems for monolingual speakers: procedural and declarative, respectively [105, 106, 111]. According to Ullman's model [111], L2 learners employ the declarative system for the learning of both types of linguistic knowledge, especially those at lower proficiency levels. That is because instead of computing morphosyntax information from smaller units in accordance with linguistic rules, such information tends to be remembered as an unanalyzed chunk for second or additional languages. We review studies that discuss L1-L2 differences in the neural aspects of morphosyntactic processing.

#### 6.3.1. Structure

The cerebellum is considered part of the procedural memory network. Its role in syntactic processing has been demonstrated in studies reporting a link between cerebellar damage and grammar impairment (see review in [147]). In their study with whole brain MRI, Pliatsikas et al. [148] report greater GM volume in several cerebellar areas for highly proficient L1 Greek/L2 English bilinguals compared with monolingual controls. Further, cerebellar GM volume was significantly correlated with behavioral performance on an English masked priming morphological task. The negative relationship between response time (i.e., faster, more efficient) and greater cerebellar volume was only evident for the L2 group, not the monolingual controls. The structure-behavior relation was also specific to a rule-based condition with past tense inflection. The conditions that did not involve rule-based morphological application did not show such a correlation, implying the cerebellum is not simply related to word reading or lexical decision tasks.

#### 6.3.2. Structural Connectivity

Most research on the connectivity of morphosyntactic language pathways for bilingual or L2 speakers shows that structural differences covary with L2 grammatical competence and learning. Using DTI, Xiang [149] found that L2 grammar competence was correlated with volume of the BA45 (pars triangularis of the IFG) to posterior temporal lobe pathway. To examine the grammar acquisition process in a more controlled way, other investigators have employed artificial language learning paradigms [150–152].

Of particular interest, Friederici et al. [150] looked at the learning of two types of syntactic information: local transitions (such as (AB)^*n*^) and hierarchical structures (such as A^*n*^B^*n*^). While the former information can be learned by both human and nonhuman primates, it is argued that hierarchical structures can only be learned by humans. This position is supported by linguistic theories which take the hierarchical nature of syntax and phonology to be the hallmark characteristic of human language (e.g., [153, 154]). Friederici et al. [150] postulated that learning of local transitional probabilities ((AB)^*n*^) can be mapped to the ventral premotor cortex and the frontal operculum (FOP) while Broca's area is responsible for the computation of complex, hierarchical information (A^*n*^B^*n*^). Participants were assigned to either of the two learning conditions with fMRI data acquired two days after learning and structural data (DTI) acquired from 4 of the participants (2 from each learning group). The authors found that the “local transition” participants showed structural connectivity of the FOP via the fasciculus uncinatus to the temporal lobe. The “hierarchical structure” participants demonstrated the same profile but showed an additional connectivity of Broca's area via the fasciculus longitudinalis superior to the temporal lobe.

Flöel et al. [151] found similar results: participants learning an artificial grammar showed a correlation between grammaticality judgment and white matter integrity in fibers originating from Broca's area. In contrast, Loui et al. [152] posited that R rather than L hemisphere areas implicated in pitch-based grammar learning. Specifically, their study showed that participants' ability to generalize learned rules to novel sequences correlated with the volume of the ventral arcuate fasciculus in the R hemisphere and with white matter integrity underlying the R temporal-parietal junction.

#### 6.3.3. Function

ERP research on the temporal dynamics of language processing yields primary neurolinguistic evidence bearing on L1-L2 syntactic processing differences especially in relation to the D/P model. Of interest are the ELAN (early L anterior negativity) and P600 effects, where the former has been interpreted as reflecting first-pass, automatic parsing, characteristic of native language processing, and the latter reflects a more controlled process of grammatical reanalysis and repair. Here, it has often been found that low-proficiency L2 learners do not evidence ELAN in syntactic/morphological violation judgments (e.g., [155–158]). For such speakers, only the less automatic/more controlled pattern of P600 effect was observed (e.g., [159, 160]). A biphasic pattern of ELAN followed by P600 is only observed in higher-proficiency speakers [160–162].

fMRI studies, in contrast with ERP studies, generally reveal that native and L2 syntactic processing recruit the same or similar regions, indicating a universal language network for syntax. Reported L1-L2 differences in such studies involve the relative degree of activation of these common areas. For instance, in a covert/silent sentence production task administered to native French speakers with moderate proficiency in English, Golestani et al. [163] found that regions such as Broca's area, dorsolateral prefrontal cortex, and R superior parietal cortex were activated in both L1 (French) and L2 (English) but that production in L2 resulted in greater activation in the L prefrontal area.

Unlike the production task in Golestani et al. [163], Wartenburger et al. [54] administered a grammaticality judgment task requiring comprehension to Italian-German bilinguals. For their early, high proficiency group, no differences were detected in brain activation regardless of whether such participants were judging sentences in their L1 or L2. The other lower proficiency groups (one early exposure and one late exposure group) showed more extensive activation involving Broca's region and subcortical areas when processing grammar in L2 versus in L1.

Also utilizing a sentence comprehension task, but varying the level of syntactic complexity, Suh et al. [164] found that processing in either L1 (Korean) or L2 (English) activated mainly the same areas, including the L inferior frontal gyrus (IFG), bilateral inferior parietal gyrus, and occipital lobe including cuneus and lingual gyrus. However, there was an effect of syntactic complexity: more complex structure induced greater activation in the L inferior frontal gyrus when processing L1 but not L2 sentences. Other studies generally support the view of shared cerebral regions for L1/L2 syntactic processing by more proficient learners [52, 165, 166].

Further, the generalization regarding a common syntactic network seems to hold true for low-proficiency L2 learners as well (e.g., [167–169]). For instance, Indefrey et al. [169] administered a grammaticality judgment task to Chinese immigrants in Netherlands after 3, 6, and 9 months of classroom learning of Dutch and found that as early as at 6 months, these L2 learners were shown to recruit areas related to native syntactic processing such as the L inferior frontal gyrus. This is somewhat problematic for the D/P model, since proceduralization and recruitment of L1-like syntax processing areas (such as inferior frontal cortex) is not predicted for beginning and low-proficiency learners.

However, not all findings support the shared network hypothesis, as different areas are found to be activated when processing L1 and L2 for certain types of morphosyntactic tasks. For instance, while Golestani et al. [163] found overlap in activation areas (noted above), covert language production in L2 English, but not in L1 French, activated the L inferior and superior parietal cortices, the R occipital cortex, and the cerebellum. On the other hand, the L putamen was found to be activated in L1 French production only. Thus, syntax may best be considered not as a monolithic module (as is the case for the D/P model) but rather as a set of more fine-grained processes as reviewed in theoretical syntax. Accordingly, contradictory findings may not be surprising given the different methodologies and tasks used in the field.

#### 6.3.4. Functional Connectivity

Dodel et al. [168] investigated “conditional dependent functional interactions” by looking at subject-dependent variables in a group of L1 French/L2 English bilinguals engaged in covert language production tasks (both lexical and syntactic). Findings of note include a more functionally linked network during L2 sentence production than during L1 consisting of the L inferior frontal gyrus, putamen, insula, precentral gyrus, and supplementary motor area. This finding held for participants with higher L2 syntactic proficiency than for those with lower proficiency.

**Figure 3:**
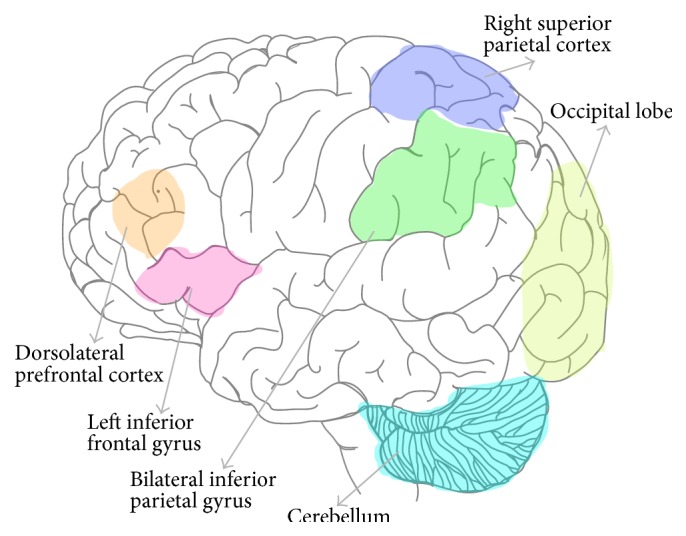
Brain areas showing variation in structure and functional activity for bilinguals performing syntactic processing tasks.

In sum, bilingual individuals had more gray matter volume than monolinguals in areas related to syntactic processing and procedural representation in the cerebellum. Differences were also reported for syntactic processing of bilinguals' L2 compared with their L1, including engagement of L inferior frontal gyrus (IFG), precentral gyrus and SMA, putamen, and SMA. Further, the degree of grammatical proficiency in one's L2 or a learned artificial grammar also corresponded to increased activity in brain areas of overlap, notably Broca's area (BA44) and connecting fibers with Broca's such as the superior longitudinal fasciculus. Structural connectivity involving Broca's area has been demonstrated for L1 learning and processing [170]. Additionally, higher L2 grammatical proficiency corresponded with electrophysiological patterns related to automatic language processing (ELAN) and cerebellar volume. The key brain areas showing variation in structure and functional activity for bilinguals performing L2 syntactic processing tasks are illustrated in Figure 3.

For morphosyntactic processing, the L1-L2 overlap seems less complete compared to lexical semantics. The wider degree of L1-L2 neurolinguistic difference lends at least partial support to the Declarative/Procedural model [111] and captures the insight on a very broad level of other similar (psycho)linguistic models such as the Fundamental Difference Hypothesis [171], Shallow Tree Hypothesis [172], and the Representational Deficit model [173] that point to divergences in representation and processing in L1 versus L2.

One issue to highlight is that the bulk of current morphosyntactic research may be beset by a “Granularity Mismatch Problem (GMP)” [174]. Specifically, the level at which linguistic computation is posed to take place is more fine-grained than the broader conceptual distinctions that form the basis of neuroscientific studies of language. For example, syntactic details concerning phrase structure, movement, and feature checking are central to linguistic theories but have no visible reflexes in current imaging data. With regard to bilingual syntax, a worthwhile pursuit would emphasize differences between “local” and “nonlocal” syntactic properties, where the latter proves more difficult for L2/bilingual learners behaviorally [172, 175, 176].

### 6.4. Effects of AoA and Proficiency

In this last section, we explore the effects of age of acquisition (henceforth AoA) and proficiency of L2/nondominant language on the location, interconnections, and intensity of activation in the bilingual brain. We also make a distinction between proficiency and task performance [162]. We refer to “proficiency” as baseline/entering/general language competence prior to the study, whereas “task performance” refers to participants' performance in a specific language task (e.g., lexical decision or grammar judgment) in studies that investigate a specific aspect of language processing. These two variables are often considered to be related (e.g., [177]). Indeed, the well-known Critical Period Hypothesis (e.g., [178]) postulates a direct correlation between AoA and ultimate attainment in native-like proficiency within a maturationally constrained time period (e.g., puberty). Therefore it would be important to attempt to disentangle these two factors in understanding how they impact the neural aspects of bilingualism (e.g., [87]). It should be noted that while some of the studies summarized and reviewed here do control for one or the other variable, others examine one factor without controlling for the other. Both types of studies are included here.

A further distinction is made between the structural and functional imaging studies in the effect of these variables. For structural imaging studies, we mainly look at how AoA, proficiency, and performance are related to GM density and WM integrity. For functional imaging studies, we mainly look at how AoA as well as proficiency further modulates the relationship between task performance and brain activation.

#### 6.4.1. Proficiency

*Structural studies* generally report a positive relationship between task performance and indicators of brain structure like fractional anisotropy and grey matter (e.g., [31, 33, 148]). For example, in Cummine and Boliek's [31] structural connectivity study, Chinese-English bilingual participants' reading performance was found to be positively associated with mean fractional anisotropy values in the parietal-occipital sulcus. In grammar processing, Greek-English bilinguals who performed better in an inflectional processing task were found to have more grey matter in the cerebellum [148]. The same pattern was observed in studies that examine the effect of general language proficiency. Mechelli et al. [83] found that, for their Italian-English bilinguals with varying AoA (2–34), those identified as having high proficiency in English (regardless of AoA) showed more grey matter density in the L inferior parietal region.

In* functional studies*, better task performance is associated with more L1-L2 similarity in functional activation. In a study where participants who all had late AoA listened to stories in different languages while undergoing PET, it was found that high proficiency Italian-English bilinguals activated similar L hemispheric areas (L temporal pole, the superior temporal sulcus, middle temporal gyrus, and hippocampal structures) whether the stories were in L1 or L2 [43]. In contrast, low proficiency bilinguals showed no activation for L2 English in those regions.

Wartenburger et al. [54] whose study is reported below for AoA effects on grammar processing also examined semantic processing (e.g.,* the deer shoots the hunter*). They found this domain of language to be affected by proficiency, but with mixed results: low proficiency L2 speakers showed more activation in Broca's area and the R middle frontal gyrus than high proficiency speakers who in turn showed more activation in the L middle frontal and R fusiform gyrus.

Proficiency also seems to be a more important factor in understanding the neural substrates of lexical processing. Chee et al. [41] examined both early and late Chinese-English bilinguals (<6 yo, >12 yo). Using fMRI, the authors found both groups activated the same areas for both L1 and L2 languages, including the prefrontal, temporal, parietal, and supplementary motor area. There was only a difference in the magnitude of activation for L1 versus L2.

ERP studies, on the other hand, provide evidence that the effect of proficiency depends on the specific language domain in question, as discussed in the section on morphosyntactic processing. While semantic anomalies elicit the similar responses (i.e., N400) in all groups (native controls, low and high proficiency groups) (e.g., [160]), syntactic violation elicits a native-like biphasic pattern of ELAN followed by P600 only in high proficiency learners (and native controls), whether in an artificial language learning paradigm (e.g., [161]) or in natural language learning cases (e.g., [160, 162, 179]).

#### 6.4.2. Age of Acquisition

In general,* structural* imaging results show that AoA is* negatively* associated with grey matter density and white matter integrity [83, 139, 180]. That is, individuals acquiring the second language early in life show increased volumetric changes (grey and white) in the brain. For example, in Mechelli et al. [83], early English-Italian bilinguals (L2 learned before 5 years of age) showed greater increase in grey matter density in the bilateral inferior parietal cortex than late bilinguals (L2 learned between 10 and 15 years of age). In Grogan et al. [139], grey matter density in another area, L pars opercularis, was negatively related to L2 AoA. For structural connection studies, Mohades et al. [180] found that simultaneous bilinguals (considered to have comparatively early AoA) showed higher FA value in the L inferior occipitofrontal fasciculus (IIFOF) than sequential bilinguals (and monolinguals). Such studies therefore seem to provide neuroimaging evidence for the maturational effects in language learning that have been observed in behavioral research.

*Functional* studies on the other hand present a more mixed picture regarding the effect of AoA, possibly due to the specific aspects of linguistic processing involved in the studies [162]. Studies suggest that AoA* positively* modifies brain activation in grammar processing, meaning that the later a second language is acquired, the more the activation is required/observed. For instance, in Wartenburger et al. [54], while early L1 Italian/L2 German learners (L2 = birth) showed no L1-L2 differences in activation, the late groups (L2 = 19 and 20 years), regardless of their proficiency, showed significantly more activation in Broca's area and subcortical structure when processing L2 grammar. Hernandez et al. [166] likewise found more neural activity in the LIFT 44/45 in later L2 Spanish learners compared with early ones while performing a grammatical gender decision task. Similar patterns were observed in Jasinska and Petitto [181] in L2 syntactic processing, for the classic language neural areas.

On the other hand, L2 AoA appears to* negatively* modify brain activation in reading and phonological processing, indicating that early AoA is related to greater activation. For example, Krizman et al. [182] found that simultaneous bilinguals (early AoA) showed greater amplitude in the auditory brainstem and more consistency in responses to synthesized syllables. In a passage reading task administered to Hindi-English bilinguals, Das et al. [57] found that L2 AoA was negatively related to L inferior parietal lobe activity. Lastly, Archila-Suerte et al.'s [183] study on phonological processing in English-Spanish bilinguals showed mixed results: While later bilinguals were found to show more neural activity than early bilinguals in the bilateral superior temporal gyrus (related to perceptual auditory information) and the Rolandic operculum (related to subvocal rehearsal), indicating a positive relationship between AoA and neural activity, the reverse was true for activity in the R middle frontal gyrus (related to high-order executive function and cognitive control). The authors explain this difference in terms of the unique linguistic environment of bilinguals (who need to manipulate speech sounds from an early age) and how that affects the allocation of brain areas for processing language information.

Finally, we consider the mode of learning by early versus late learners, and how such a difference may account for some of AoA effects reported in the studies reviewed above. It has long been hypothesized in the second language acquisition literature that, unlike monolingual/young learners who learn certain language aspects implicitly (without awareness of what is being learned), late learners adopt a more explicit approach [184, 185] whereby they notice negative evidence and make use of pedagogical grammatical descriptions and analogical reasoning, among other things [185].

In cognitive psychology, there are further proposals mapping these learning modes to different language domains (speech/phonology, syntax, lexical semantics, etc.) and different types of language competence (e.g., “Basic Interpersonal Communication Skills” (BICS) versus “Cognitive and Language Proficiency” (CALP), [186]). Various researchers have associated early (implicit) learning with grammar (e.g., [105, 106]) and speech sounds (e.g., [187]), in contrast with (lexical) semantics which characterize late, explicit learning. Consequently, late learners might adopt a different approach (perhaps more conscious, effortful, and academic-like) to learning language (aspects) than early learners (e.g., [188]).

Some of the studies reported here can be interpreted in terms of such a model. For instance, Wartenburger et al.'s [54] study found an effect of AoA for grammar processing. It could be that if rule-based knowledge such as grammar is learned at a later stage and via the explicit mode, a different pattern of neural recruitment is observed when processing L2 (e.g., increased intensity in activation, as was reported in the study) which could be neural reflexes of effortful learning. The absence of L1-L2 activation difference for grammar processing in the early group is then perhaps reflective of the absence of such conscious, effortful learning for L2ers.

Likewise, Archila-Suerte et al.'s [183] finding that late bilinguals showed increased activity in the bilateral rolandic operculum when processing L2 speech (as compared with early bilinguals and monolinguals) could also be explained by the differences in the mode of learning. This premotor area has been linked to subvocal rehearsal, which, as the authors point out, is important for L2ers for whom the interconnection of L2 sounds may be less strong than L1 sounds. Therefore, the more effortful learning of L2 speech may be reflected in the increased activation of this brain region. A summary of the studies reviewed in this section is shown in Table 1.

## 7. Conclusion

We have systematically reviewed studies that employ advanced neuroimaging techniques to study the impact of bilingualism on brain structure, structural connectivity, function, and functional connectivity. The first issue we addressed, whether the language neural network is different for L1 versus L2 processing, revealed evidence that similar brain networks are activated for L1 and L2 in the domains of reading, listening, and speech production. Secondly, on the effects of bilinguals' executive functioning on the structure and function of the “universal” language neural network, the reviewed studies indicate that stronger cognitive control in bilinguals is accompanied by increased gray and white matter volume and regional activation in the frontoparietal network and basal ganglia.

The third issue on the effects of bilingualism on phonological, lexical-semantic, and syntactic aspects of language processing conveyed that bilinguals generally showed increased volume in component language structures and the connective tracts between these brain areas compared to monolinguals. Further, the degree of convergence/divergence in brain regions and networks involved in L1 and L2 processing is related to the linguistic processes involved. Specifically, the largest degree of divergence in structure, function, and/or connectivity is observed in phonology, followed by morphosyntax and semantics. It is likely that the development of these brain regions may parallel language developmental milestones, with phonological development beginning first, followed by semantic development, and finally grammatical/syntax development. In line with the often observed difference in reliability and convergence in language systems (between first and second language acquisition, e.g., Fundamental Difference Hypothesis [112]), sensitive periods do not apply to language broadly, rather different linguistic domains or components are affected in a nonuniform manner, with phonology being most susceptible to age effects and syntax to a lesser degree (around adolescence), while vocabulary has no age constraints at all (e.g., [162, 189]).

With regard to the fourth issue, we found that factors such as age of acquisition and proficiency levels further modify the location, interconnections, and intensity of activation in the bilingual brain, especially when considered with respect to the different component processes. Studies indicate that, generally, the earlier a language is learned and the higher proficiency is attained in L2, the more the grey matter intensity and white matter integrity are observed. Functional results, on the other hand, seem to depend on the specific nature of the component processes. While phonology and syntactic knowledge are generally more sensitive to age effects (earlier AoA = less activation), lexical semantics, on the other hand, is more affected by proficiency levels (higher proficiency = more L1-like activation, generally).

In interpreting the nascent neurolinguistics literature, methodological differences between investigations should be taken into account [73], but at the same time advancement in this scientific area would also benefit from multiple sources of information [190]. Therefore, in this review we included findings from research employing diverse neuroscience methods and we considered their concurrence in light of current cognitive and linguistic models. We did not find obvious alignment of structure and function connectivity within the area of neurolinguistics, but we are optimistic that current methodologies emphasizing dynamic and emergent neural networks can supplement behavioral research to inform bilingual models [191].

There are a number of limitations to the conclusions that can be drawn at present. For instance, we note here that not all studies investigating language proficiency controlled for AoA, and vice versa. Thus, future studies should consider the possibility of holding all other language-related variables constant. Future studies may also consider investigating the factor of AoA longitudinally, by following a population of bilingual children across developmental time, or cross-sectionally, by studying bilingual children of varying age ranges at a single time point.

Other considerations for future research regard the mismatch of “granularity” between the disciplines of linguistics and the neuroscience of language. In the neuroscience of language, the terms “phonology, semantics, and syntax” are used in a very general sense to refer to “sound structure, word meaning, and phrase structure,” whereas in contemporary linguistics, each of those subfields necessarily consists of numerous computations and much finer-grained representations [174]. In addition, current understanding of linguistics often emphasizes the interconnections or interfaces of different linguistic submodules as well as nonlinguistic information, instead of treating them as separate, isolated domains [192].

Future consideration of neural correlates, especially in terms of connectivity, could focus on issues like how linguistic interfaces affect completeness of bilingual acquisition and L1 attrition (e.g., [193, 194]). Finally, additional factors not included in our review may also prove relevant in the neurolinguistics of bilingualism, such as language types with two typologically/phonetically similar languages (e.g., German and English), as compared to bilinguals with two typologically/phonetically dissimilar languages (e.g., Tamil and English). While this goes beyond the scope of the current review, future work may wish to consider investigating the impact of different language types/pairings on the bilingual brain.

## Figures and Tables

**Table 1:** Summary of studies reviewed as related to the variables of age of acquisition and proficiency.

Reference	Nature of task (lexical/phonological/syntactical)	Group differences (age of acquisition/language proficiency/task performance)	Methodology and structural/functional effects on the brain	Details
Mechelli et al., 2004, Nature [83]	—	L2 proficiency (indexed by neuropsychological tests of reading, writing, speech comprehension, and production) AoA (monolinguals versus early bilinguals versus late bilinguals)	Structure: VBM (grey matter density)	L2 proficiency was positively related to grey matter density in L inferior parietal cortex (and negatively related to AoA) L2 AoA was negatively related to grey matter density in L and R inferior parietal cortex

Klein et al., 2014, Brain and Language [123]	—	AoA (simultaneous versus sequential early versus sequential late bilinguals)	Structure: MRI (cortical thickness)	L2 AoA was associated with cortical thickness; positive relationship for L inferior frontal gyrus and L superior parietal lobe, negative relationship for R inferior frontal gyrus The researchers argued that the positive relationship could reflect the growth of new cortical tissue as demanded by the new learning in adolescence/adulthood and the ensuing cortical folding to accommodate new tissue

Mohades et al., 2012, Brain Research [180]	—	AoA (simultaneous bilinguals versus sequential bilinguals versus monolinguals)	Structure: MRI DTI (white matter)	L2 AoA was negatively associated with mean fractional anisotropy values for inferior occipitofrontal fasciculus (IIFOF) tracts negatively associated with AoA L2 AoA was positively associated with mean fractional anisotropy values for the bundle of white matter fibres arising from the anterior part of the corpus callosum projecting to the orbital lobe (AC-OL)

Grogan et al., 2012, Neuropsychologia [139]	Lexical (efficiency)	Task performance (speed and accuracy of lexical decisions, and lexical fluency) AoA	Structure: MRI/VBM	L2 task performance was negatively related to grey matter in the L pars opercularis L2 AoA was negatively related to grey matter in the L pars opercularis

Pliatsikas et al., 2014, Cerebellum [148]	Lexical (grammar)	Task performance (speed of processing regular inflections) Years of naturalistic exposure (number of years living in UK)	Structure: VBM	L2 task performance was positively associated with grey matter volume in the cerebellum Years of naturalistic exposure to L2 were positively correlated with grey matter volume in the posterior bilateral putamen

Das et al., 2011, Neuroimage [57]	Lexical (reading proficiency)	Task performance AoA (simultaneous versus sequential bilinguals)	Function: fMRI	L2 task performance (English reading proficiency) was positively related to L inferior temporal gyrus activity L2 AoA was negatively related to left inferior parietal lobe activity

Cummine and Boliek, 2013, Brain Structure and Function [31]	Lexical (reading)	Task performance (response time for a complex reading task) for sequential bilinguals versus monolinguals	Structure: DTI (white matter)	L2 task performance was positively associated with mean fractional anisotropy values in the parietal-occipital sulcus The researchers contended that the parietal-occipital sulcus could be a possible gating system for modulating the contribution of sublexical and lexical stream processes

Grogan et al., 2009, Cerebral Cortex [33]	Lexical + phonological	Task performance (semantic and phonemic fluency scores)	Structure: MRI, VBM	Task performance (phonemic fluency) in L1 and L2 was associated with grey matter in the presupplementary motor area and the head of caudate Task performance (semantic fluency) in L1 and L2 was positively associated with grey matter density in the L inferior temporal cortex

Krizman et al., 2015, Neuroscience Letters [182]	Phonological (listening to syllables)	AoA, matched for proficiency (simultaneous versus sequential)	Function: EEG	L2 AoA was negatively associated with amplitude of fundamental frequency response in the auditory brainstem to syllables like “ba” and “ga” L2 AoA was negatively associated with neural consistency of responses to syllable “ba” The researchers argued that bilingualism enhances subcortical auditory processing

Archila-Suerte et al., 2015, Brain and Language [183]	Phonological (processing L2 speech sounds)	AoA (early versus late), matched for proficiency and SES	Function: fMRI	L2 AoA was positively associated with neural activity in the bilateral superior temporal gyrus and the Rolandic operculum The researchers posited that late bilinguals recruit this premotor area to support subvocal rehearsal of L2 sounds learned late in development L2 AoA negatively associated with neural activity in the R middle frontal gyrus

Golestani et al., 2006, Neuropsychologia [163]	Syntax	L2 grammatical (syntactic) proficiency as assessed with TOEFL in late bilinguals	Function: fMRI	L2 task proficiency was positively associated with neural activity in the basal ganglia (particularly left caudate nucleus/putamen) During syntactical production, language proficiency was positively related to the closeness of L1 and L2 activity peaks in L inferior frontal gyrus

Jasinska and Petitto, 2013, Developmental Cognitive Neuroscience [181]	Syntactical (sentence judgment task)	AoA (early versus late)	Function: fNIRS	In children, L2 AoA was positively associated with neural activity in the classic language neural areas (bilateral superior temporal gyrus, Broca's area) and negatively associated with neural activity in domain-general areas (dorsolateral prefrontal cortex, frontopolar cortex)
